# The effects of oxaliplatin-based adjuvant chemotherapy in high-risk stage II colon cancer with mismatch repair-deficient: a retrospective study

**DOI:** 10.1186/s12885-024-11821-w

**Published:** 2024-02-02

**Authors:** Leen Liao, Jinghua Tang, Zhigang Hong, Wu Jiang, Yuan Li, Lingheng Kong, Kai Han, Zhenlin Hou, Chenzhi Zhang, Chi Zhou, Linjie Zhang, Qiaoqi Sui, Binyi Xiao, Weijian Mei, Jiehai Yu, Wanjun Yang, Zhizhong Pan, Pei-Rong Ding

**Affiliations:** 1 Department of Colorectal Surgery, Sun Yat-Sen University Cancer Center, Guangzhou, China; 2 State Key Laboratory of Oncology in South China, Guangdong Provincial Clinical Research Center for Cancer, Sun Yat-sen University Cancer Center, Guangzhou, China

**Keywords:** Mismatch repair deficient, Stage II colon cancers, Adjuvant chemotherapy

## Abstract

**Background:**

For high-risk stageIImismatch repair deficient (dMMR) colon cancers, the benefit of adjuvant chemotherapy remains debatable. The principal aim of this study was to evaluate the prognostic value of high-risk factors and the effect of oxaliplatin-based adjuvant chemotherapy among dMMR stageIIcolon cancers.

**Methods:**

Patients with stage II dMMR colon cancers diagnosed between June 2011 and May 2018 were enrolled in the study. Clinicopathological characteristics, treatment, and follow-up data were retrospectively collected. The high-risk group was defined as having one of the following factors: pT4 disease, fewer than twelve lymph nodes harvested (< 12 LNs), poorly differentiated histology, perineural invasion (PNI), lymphatic vascular invasion (LVI), or elevated preoperative carcinoembryonic antigen (CEA). The low-risk group did not have any risk factors above. Factors associated with disease-free survival (DFS) were included in univariate and multivariate Cox analyses.

**Results:**

We collected a total of 262 consecutive patients with stage II dMMR colon cancer. 179 patients (68.3%) have at least one high-risk factor. With a median follow-up of 50.1 months, the low-risk group was associated with a tended to have a better 3-year DFS than the high-risk group (96.4% vs 89.4%; *P* = 0.056). Both elevated preoperative CEA (HR 2.93; 95% CI 1.26–6.82; *P* = 0.013) and pT4 disease (HR 2.58; 95% CI 1.06–6.25; *P* = 0.037) were independent risk factors of recurrence. Then, the 3-year DFS was 92.6% for the surgery alone group and 88.1% for the adjuvant chemotherapy group (HR 1.64; 95% CI 0.67–4.02; *P* = 0.280). Furthermore, no survival benefit from oxaliplatin-based adjuvant chemotherapy was observed in the high-risk group and in the subgroups with pT4 disease or < 12 LNs.

**Conclusions:**

These data suggests that not all high-risk factors have a similar impact on stage II dMMR colon cancers. Elevated preoperative CEA and pT4 tumor stage are associated with increased recurrence risk. However, oxaliplatin-based adjuvant chemotherapy shows no survival benefits in stage II dMMR colon cancers, either with or without high-risk factors.

**Supplementary Information:**

The online version contains supplementary material available at 10.1186/s12885-024-11821-w.

## Background

Adjuvant chemotherapy has been demonstrated to significantly improve 5-years overall survival (OS) by 20–33% in patients with stage III colon cancers (CCs) [1, 2]. However, its application in patients with stage II CCs remains controversial [3, 4]. Based on the currently available data, the National Comprehensive Cancer Network (NCCN) guidelines recommend chemotherapy for patients with high-risk tumor features [5, 6]. These high-risk factors include pT4 diseases, tumor obstruction or perforation, fewer than twelve lymph nodes harvested (< 12 LNs), poorly differentiated histology, perineural invasion (PNI), and lymphatic vascular invasion (LVI), or elevated preoperative carcinoembryonic antigen (CEA). However, not all high-risk factors have a similar prognostic effect [7]. Some studies suggested that pT4 tumor stage and < 12 LNs were the most relevant risk factors after surgery for colon cancers [4, 8]. Additionally, mismatch repair status serves as a highly recognized biomarker in stage II CCs [9, 10]. Mismatch repair deficiency (dMMR) is observed in approximately 10%-15% of stage II CCs, which signifies a notably low likelihood of recurrence [11, 12]. This molecular characteristic plays a crucial role in prognosis assessment and treatment decision-making for stage II CCs [13, 14].

Importantly, the interaction between dMMR status and high-risk features in stage II CCs has not been thoroughly studied and remains unclear. This relationship is significant for clinical decision-making, especially considering the conflicting guidelines regarding adjuvant chemotherapy in stage II dMMR CCs. The NCCN guidelines recommend against adjuvant chemotherapy for stage II dMMR CCs regardless of high-risk factors [7]. This recommendation is primarily based on evidence showing that dMMR cancers do not respond well to 5-fluorouracil-based adjuvant chemotherapy [15, 16]. On the other hand, the European Society for Medical Oncology (ESMO) guidelines suggest that oxaliplatin-based adjuvant chemotherapy is recommended for stage II dMMR CCs with pT4 tumor stage, < 12 LNs harvested, or multiple intermediate risk factors [8]. However, the low prevalence and good outcomes of stage II dMMR CCs limit the conclusive evidence for benefit from oxaliplatin in this population. Meanwhile, specific benefit of adjuvant chemotherapy conferred by these high-risk factors are not yet fully understood. This inherent ambiguity introduces subjectivity and variability in clinical decision-making for stage II dMMR CCs.

Selection of stage II dMMR CCs who benefit from therapeutic regimens helps to improve treatment efficacy and potentially avoid toxicity. For this purpose, we evaluated the prognostic value of high-risk factors and the efficacy of oxaliplatin-based adjuvant chemotherapy in stage II dMMR CCs.

## Patients and methods

### Study population and design

This retrospective and consecutive cohort study was conducted at Sun Yat-sen University Cancer Center, enrolling patients with stage II dMMR CCs between June 2011 and May 2018. All patients underwent curative resection with or without subsequent adjuvant chemotherapy. The screening strategy and treatment approach were depicted in Fig. S1.

High-risk factors were used to stratify patients into high-risk and low-risk groups. According to NCCN and ESMO guidelines, high-risk factors were defined as pT4 tumor stage, < 12 LNs, poorly differentiated histology, PNI, LVI, or preoperative carcinoembryonic antigen (CEA) > 5.0 ng/ml. Information on tumor obstruction, tumor perforation, or positive margins were rare in this cohort and hence were not included in the analysis. Preoperative CEA levels were assessed within one week before the operation, while postoperative CEA levels were evaluated within four weeks after the operation and prior to the initiation of adjuvant chemotherapy. Normalized postoperative CEA was elevated preoperative CEA (> 5.0 ng/mL) but normal postoperative CEA. Elevated postoperative CEA meaned that preoperative and postoperative CEA levels were both elevated (> 5.0 ng/mL).

### IHC Analysis of MMR protein expression

MMR protein (MLH1, MSH2, MSH6, and PMS2) expression was analyzed in formalin-fixed paraffin-embedded (FFPE) tumor sections. Protein expression was considered negative when nuclear staining was completely absent in neoplastic cells. The absence of nuclear staining in tumor cells and normal staining in the surrounding normal tissue was considered as dMMR. Immunohistochemical (IHC) staining was scored by an experienced gastrointestinal pathologist.

### Statistical analysis

All the categorical variables were presented with frequencies and proportions. Continuous variables were compared using the Mann–Whitney U test. Categorical variables were compared using the chi-squared test. Disease-free survival (DFS) was the primary endpoint for survival analyses. DFS was calculated from the date of surgery until the evidence of progression or the last date of follow-up and was estimated using the Kaplan–Meier method with the log-rank test. Survival data was analyzed using Cox proportional hazard regression. Only variables with P values ≤ 0.10 in univariate analysis were eligible for the multivariate Cox regression model. Statistical analyses were performed using the R software (version 4.0.2; http://www.r-project.org/) and SPSS (Version 20.0; IBM Corp, Armonk, NY, USA). All tests were two-sided and statistical significance was set at *P* < 0.05.

## Results

### Patient characteristics

Patient and disease characteristics were shown in Table 1. Between 2011 and 2018, 262 consecutive dMMR patients were identified with stage II CCs, of whom 179 patients had at least one high-risk factor; 88 (49.2%) had only one, and 91 (50.8%) had two or more high-risk factors. The median age of the patients was 54 years (range 22–85 years). Among all the patients, 61.8% were male and 38.2% were female. The common high-risk factors included poor differentiation (37.4%), elevated preoperative CEA (29.4%), pT4 tumor stage (17.6%), < 12 LNs (15.7%), and LVI (11.1%). The presence of PNI was the least common risk factor (8.0%).

**Table 1:** Univariate and multivariate analysis of 3-DFS in stage II dMMR patients

**Variables**	**N (%)**	**Univariate Analysis**		**Multivariate Analysis**	
		**HR (95% CI)**	***P*-value**	**HR (95% CI)**	***P*-value**
Age (years)					
≥ 65	53(20.2)	1.16(0.43–3.15)	0.769		
< 65	209(79.8)				
Gender					
Male	162(61.8)	1.35(0.55–3.32)	0.508		
Female	100(38.2)				
Family history					
Yes	110(42.0)	1.14(0.49–2.64)	0.762		
No	152(58.0)				
pT4 category					
Yes	46(17.6)	2.93(1.23–6.98)	0.015	2.58(1.06–6.25)	0.037
No	216(82.4)				
Lymphatic node number					
< 12 LNs	41(15.7)	2.16(0.85–5.53)	0.107		
≥ 12 LNs	221(84.3)				
LVI					
Yes	29(11.1)	2.54(0.94–6.89)	0.067	1.71(0.60–4.89)	0.316
No	233(88.9)				
PNI					
Yes	21(8.0)	1.18(0.28–5.04)	0.830		
No	241(92.0)				
Histological grade					
Poorly differentiated	98(37.4)	2.08(0.90–4.82)	0.087	1.65(0.68–4.02)	0.270
Moderately differentiated	164(62.6)				
Pre-CEA					
Abnormal (> 5 ng/ml)	77(29.4)	3.08(1.33–7.12)	0.009	2.93(1.26–6.82)	0.013
Normal	185(80.6)				
High-risk Factor					
Yes	179(68.3)	3.08(0.91–10.41)	0.056		
No	83(31.7)				
Multiple risk Factor					
Yes	91(34.7)	2.88(1.23–6.74)	0.015		
No	171(65.3)				

*Abbreviations*: < *12 LNs* Fewer than twelve lymph nodes harvested, *LVI* Lymphatic vascular invasion, *PNI* Perineural invasion, *Pre-CEA* Preoperative carcinoembryonic antigen *HR* Hazard ratio

### Survival according to risk factors

**Fig. 1:**
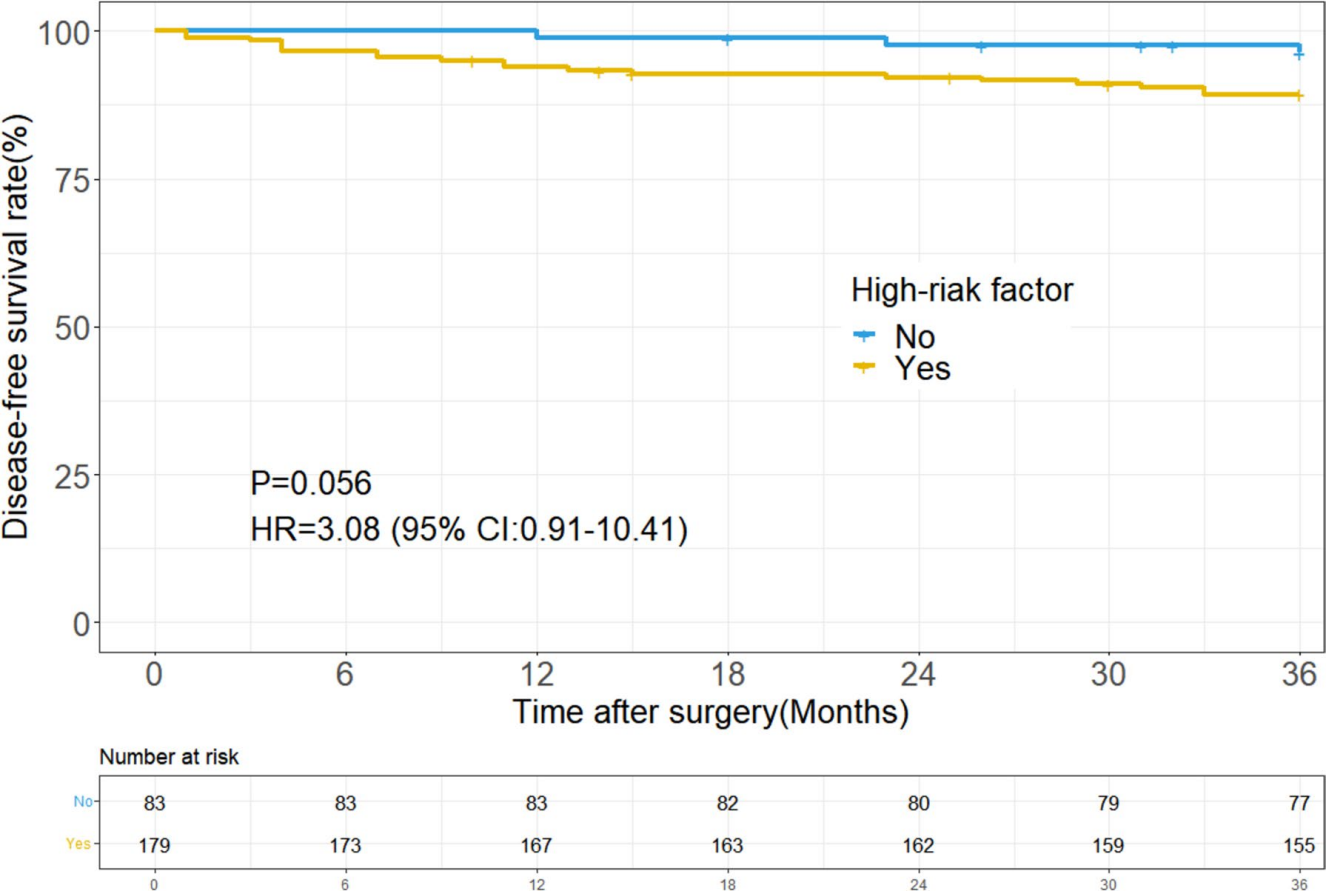
Disease-free survival in patients with stage II dMMR colon cancers by high-risk factor status

**Fig. 2:**
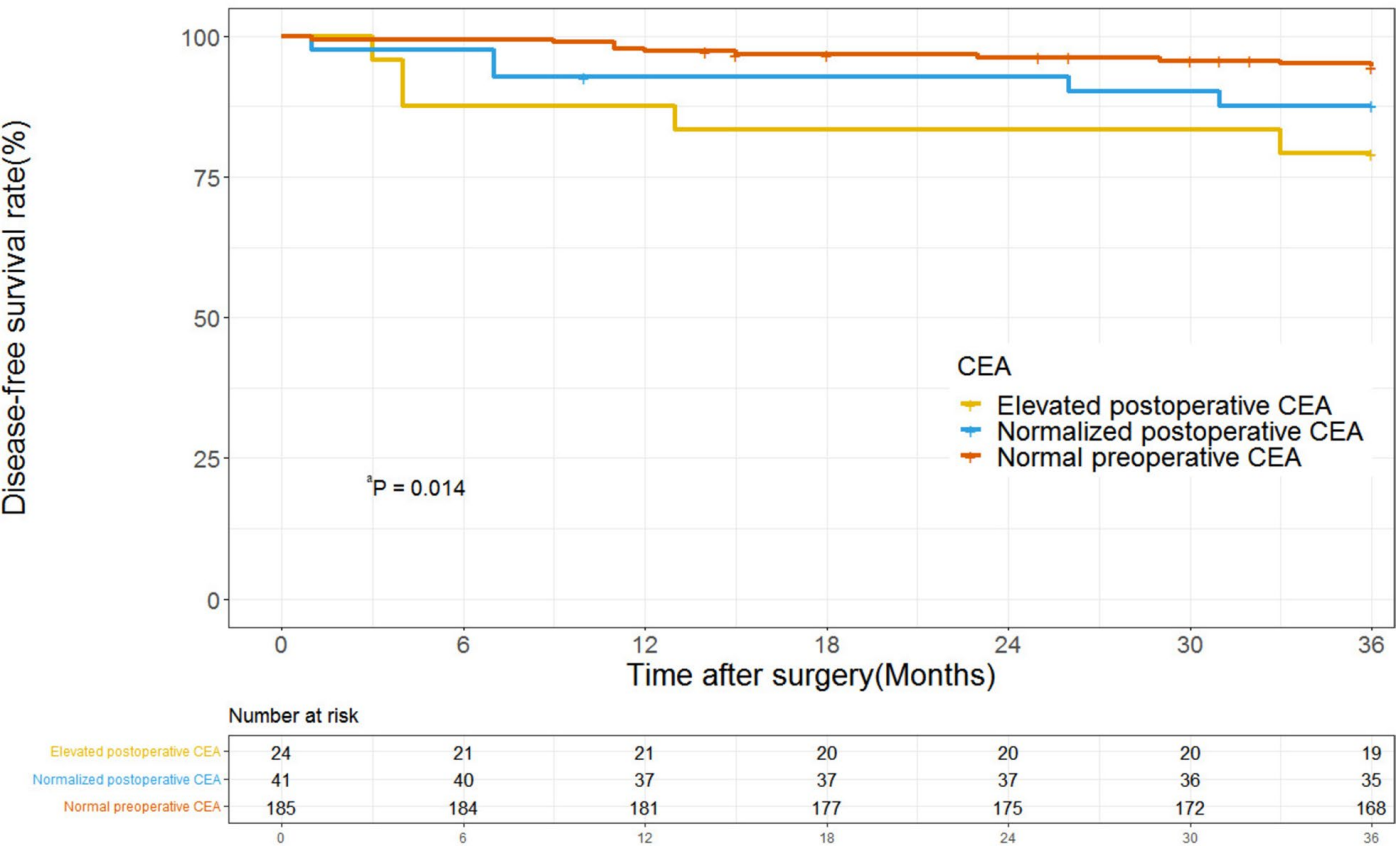
Disease-free survival in patients with stage II dMMR colon cancers by CEA status. ^**a**^ Normal preoperative CEA vs elevated postoperative CEA (HR 4.25; 95%CI, 1.44–12.43; *P* = 0.008); Normal preoperative CEA vs normalized postoperative CEA,(HR 2.37; 95% CI, 0.81–6.94; *P* = 0.115)

With a median follow-up of 50.1 months, the low-risk group tended to have a better 3-year DFS than the high-risk group (96.4% vs 89.4%; *P* = 0.056; Fig. 1). On univariate analysis, pT4 tumor stage (HR 2.93; 95% CI 1.23–6.98; *P* = 0.015) and elevated preoperative CEA (HR 3.08; 95% CI 1.33–7.12; *P* = 0.009) were associated with poor DFS (Table 1). In the multivariate Cox regression model, pT4 tumor stage (HR 2.58; 95% CI 1.06–6.25; *P* = 0.037) and elevated preoperative CEA (HR 2.93; 95% CI 1.26–6.82; *P* = 0.013) still were the independent prognostic factors of DFS, respectively. The 3-year DFS was 84.4% (95% CI, 74.7%-90.1%) in the patients with high preoperative CEA compared with 94.6% (95% CI, 90.3%-97.0%) in the patients with normal preoperative CEA. Among the 77 patients with elevated preoperative CEA, 65 (84.4%) had postoperative CEA data available; 41 (63%) of these patients had normalized postoperative CEA levels and 24 (37%) had elevated postoperative CEA levels. The overall 3-year DFS was 87.8% (95% CI, 74.4%-94.7%) in the 41 patients with normalized postoperative CEA, which was not statistically distinguishable from the 94.6% (95% CI, 90.3%-97.0%) in the patients who had normal preoperative CEA levels (HR 2.37; 95% CI, 0.81–6.94; *P* = 0.115). However, the 3-year DFS was significantly lower in the elevated postoperative CEA group than in the normal preoperative CEA groups (79.2% vs 94.6%; HR 4.25; 95%CI, 1.44–12.43; *P* = 0.008) (Fig. 2).

### Association between risk factors and adjuvant therapy

In total, 203 (77.5%) patients underwent surgery alone and 59 (22.5%) patients received surgery and oxaliplatin-based adjuvant chemotherapy. There were 49 (27.4%) patients and 10 (12.0%) patients who received treatment in the high-risk and low-risk groups, respectively. Patients were more likely to receive oxaliplatin-based adjuvant chemotherapy because of high-risk factors (*P* = 0.006), such as pT4 tumor stage (40.7% vs 10.8%; *P* < 0.001), PNI (22.0% vs 3.9%; *P* < 0.001) and LVI (20.3% vs 8.4%; *P* < 0.01). Additional characteristics were described in Fig. S2.

### Survival according to risk factors and adjuvant therapy

**Fig. 3:**
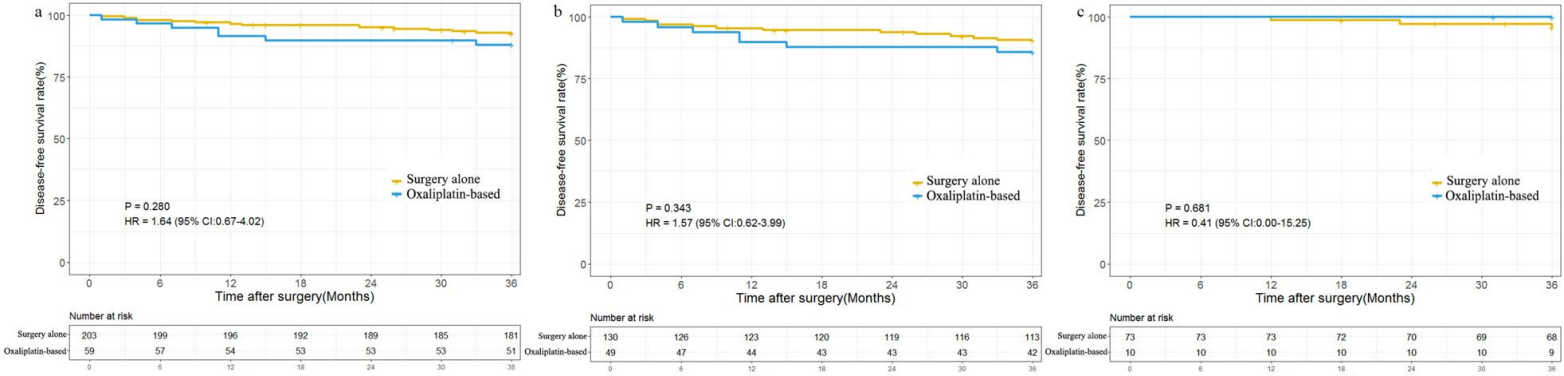
Kaplan–Meier plots showing outcome in patients with stage II dMMR colon cancers according to treatment and high-risk status. **a** Disease-free survival (DFS) in all patients. **b** DFS in highrisk group. **c** DFS in lowrisk group

The 3-year DFS was 92.6% and 88.1% in the surgery alone and adjuvant chemotherapy group (HR 1.64; 95% CI 0.67–4.02; *P* = 0.280; Fig. 3a), respectively. No significant treatment benefit was observed in stage II CCs patients with dMMR in either high-risk group (HR 1.57; 95% CI, 0.62 to 3.99; *P* = 0.343; Fig. 3b) or low-risk group (HR 0.41; 95% CI, 0.00 to 15.25; *P* = 0.681; Fig. 3c). Given that ESMO guideline suggesting pT4 tumor stage and < 12 LNs were major high-risk factors in stage II CCs, so subgroup analyses were conducted. The two subgroups showed similar demographics and clinicopathologic characteristics (Tables 2 and 3). However, no potential benefit from oxaliplatin-based chemotherapy was observed in either subgroup (Fig. S3).

**Table 2:** Clinicopathologic characteristics of pT4 patients stratified by treatment

**Population characteristics**	**Surgery alone** **(*N* = 22)**	**Oxaliplatin-based** **(*N* = 24)**	***P-*value**
Age (years)			0.052
≥ 65	8(36.4)	2(8.3)	
< 65	14(63.6)	22(91.7)	
Gender			0.840
Male	4(18.2)	6(25.0)	
Female	18(81.8)	18(75.0)	
Family history			1.000
Yes	11(50.0)	12(50.0)	
No	11(50.0)	12(50.0)	
Tumor site			0.416
Right colon	13(59.1)	12(50.0)	
Left colon	8(36.4)	8(33.3)	
Multiple primary colorectal	1(4.5)	4(16.7)	
Lymphatic node number			0.402
< 12 LNs	10(45.5)	7(29.2)	
≥ 12 LNs	12(54.5)	17(70.8)	
LVI			0.129
Yes	1(4.5)	6(25.0)	
No	21(95.5)	18(75.0)	
PNI			0.900
Yes	4(18.2)	3(12.5)	
No	18(81.8)	21(87.5)	
Histological grade			
Poorly differentiated	12(54.5)	12(50.0)	0.990
Moderately differentiated	10(45.5)	12(50.0)	
Pre-CEA			0.606
Abnormal (> 5 ng/ml)	8(36.4)	6(25.0)	
Normal	14(63.6)	18(75.0)	
Multiple risk factors			0.702
Yes	19 (86.4)	19 (79.2)	
No	3(13.6)	5 (20.8)	

*Abbreviations*: < *12 LNs* Fewer than twelve lymph nodes harvested, *LVI* Lymphatic vascular invasion, *PNI* Perineural invasion, *Pre-CEA* Preoperative carcinoembryonic antigen

**Table 3:** Clinicopathologic characteristics of patients with < 12 LNs stratified by treatment

**Population characteristics**	**Surgery alone** **(*N* = 29)**	**Oxaliplatin-based** **(*N* = 12)**	***P*** **value**
Age (years)			0.336
≥ 65	11(37.9)	2(16.7)	
< 65	18(62.1)	10(83.3)	
Gender			0.254
Male	9(31.0)	1(8.3)	
Female	20(69.0)	11(91.7)	
Family history			1.000
Yes	10(34.5)	4(33.3)	
No	19(65.5)	8(66.7)	
Tumor site			0.755
Right colon	13(44.8)	4(33.3)	
Left colon	11(37.9)	5(41.7)	
Multiple primary colorectal	5(17.2)	3(25.0)	
pT4 category			0.288
Yes	10(34.5)	7(58.3)	
No	19(65.5)	5(41.7)	
LVI			0.969
Yes	3(10.3)	2(16.7)	
No	26(89.7)	10(83.3)	
PNI			1.000
Yes	5(17.2)	2(16.7)	
No	24(82.8)	10(83.3)	
Histological grade			0.938
Poorly differentiated	10(34.5)	5(41.7)	
Moderately differentiated	19(65.5)	7 (58.3)	
Pre-CEA			0.577
Abnormal (> 5 ng/ml)	9(31.0)	2(16.7)	
Normal	20(69.0)	10(83.3)	
Multiple risk feature			0.183
Yes	19(65.5)	11(91.7)	
No	10(34.5)	1(8.3)	

*Abbreviations* < *12 LNs* Fewer than twelve lymph nodes harvested, *LVI* Lymphatic vascular invasion, *PNI* Perineural invasion, *Pre-CEA* Preoperative carcinoembryonic antigen

## Discussion

Our study demonstrates that the conventional high-risk factors have different impacts on the survival benefit in stage II dMMR CCs. Specifically, pT4 tumor diseases and elevated preoperative CEA levels have a more significant impact on 3-year DFS than other factors. Furthermore, oxaliplatin-based adjuvant chemotherapy may not be associated with favorable survival in patients with stage II CCs regardless of risk status.

The ESMO and NCCN clinical guidelines recommend chemotherapy for stage II CCs patients with high-risk tumor features, such as < 12 LNs, pT4 stage, LVI, high preoperative CEA, and so on [7, 8]. However, it remains unclear whether these risk factors matter similarly in the dMMR CCs [17]. Our study has shed light on this matter by indicating that the risk of recurrence in stage II CCs varies depending on the specific high-risk factor. The factor of pT4 disease has a considerable negative impact on survival (HR 2.58; 95% CI 1.06–6.25; *P* = 0.037). This result aligns with the outcomes reported by Cohen et al., who observed significantly worse outcomes associated with pT4 tumor stage in both proficient MMR (pMMR) and dMMR tumors, with a stronger effect observed in the latter group [18].

Previous research supported for using preoperative CEA as a risk factor to guide the recommendation of adjuvant treatment in stage II CCs patients [19]. Patients with elevated preoperative CEA levels had a 7.4% higher 3-year recurrence-free survival compared to those with normal preoperative CEA levels [20]. However, the applicability of these findings to dMMR CCs was uncertain due to limited data. Our data demonstrated that an elevated preoperative CEA in dMMR CCs was associated with worse outcomes compared to patients with CEA levels < 5 ng/ml (HR 2.93; 95% CI 1.26–6.82; *P* = 0.013). Similar to the previous study, we observed that more than 50% of patients with elevated preoperative CEA levels experienced normalization of CEA levels after surgery, and there was no significant difference in outcomes between patients with normal postoperative CEA levels and those with normal preoperative CEA levels (HR 2.37; 95% CI, 0.81–6.939; *P* = 0.115). Furthermore, our study highlighted the potential use of postoperative CEA in the prognostic stratification of stage II CCs. However, due to the limited number of patients (9.1%) with high postoperative CEA levels in our study, additional prospective data collection is required to validate and confirm our findings in this regard.

The ACCENT database, a comprehensive source of clinical trial data, has provided evidence that oxaliplatin-based adjuvant treatment significantly improves DFS in stage III dMMR CCs (HR = 0.52; 95% CI, 0.28 to 0.93) [18]. This finding supports the hypothesis that the sensitivity of oxaliplatin chemotherapy is independent of the MMR system, as platinum–DNA adducts generated by oxaliplatin lead to DNA-strand breaks that cannot be recognized and repaired in dMMR cells [21]. Based on this hypothesis, the ESMO guidelines recommend the use of oxaliplatin-based adjuvant therapy for selected patients with high-risk stage II dMMR CCs. However, direct evidence supporting this recommendation is lacking. In our study, no benefit could be shown in oxaliplatin-based adjuvant chemotherapy compared to surgery alone in either high-risk or low-risk stage II dMMR CCs. Consistent with our findings, previous studies have also suggested that patients with high-risk stage II dMMR CCs do not derive significant benefits from FOLFOX adjuvant therapy, regardless of the treatment course [22]. The relatively favorable prognosis of high-risk stage II dMMR CCs after surgery alone, compared to stage III CCs, may contribute to the lack of significant benefit from adjuvant therapy.

It is important to note that not all high-risk factors in stage II CCs are associated with survival benefits with adjuvant chemotherapy [23, 24]. According to the ESMO guidelines, factors such as < 12 LNs and pT4 tumor stage are important prognostic parameters for risk assessment in stage II CCs. However, in our study, despite a high percentage of patients with pT4 diseases (47.7%) and < 12 LNs examined (25.0%) receiving adjuvant chemotherapy, there was no difference in 3-year DFS observed between different treatment groups in these subgroups. One possible explanation is that an antitumor immune response characterized by lymphocytic infiltration, which is commonly observed in dMMR tumors, may be hindered by the immunosuppressive effects of chemotherapy. This could potentially diminish the efficacy of adjuvant chemotherapy in this patient population [12]. Based on our findings, it is evident that patients with stage II dMMR CCs, even in the presence of high-risk factors, do not derive significant benefits from oxaliplatin-based adjuvant chemotherapy. Therefore, alternative therapeutic strategies should be explored for this patient population. One potential approach could be the analysis of circulating tumor DNA before initiating chemotherapy, which may provide valuable information for personalized treatment decisions. Additionally, emerging immunotherapy strategies hold promise and should be further investigated for their effectiveness in stage II dMMR CCs [25–27].

There are several limitations in this study, including those inherent to a retrospective observational study design in any single institutional and observational retrospective analysis. Another limitation is the inadequate availability of information on certain clinical parameters. Specifically, data on tumor obstruction or perforation, which are important prognostic factors, were scarce and not included in the analysis. Additionally, molecular markers such as KRAS and BRAF V600E mutations, which have implications for prognosis and treatment decisions, were not extensively evaluated in our study [28, 29]. Finally, the study lacks detailed information on chemotherapy duration and toxicity, which could potentially impact the survival benefit observed.

In conclusion, our study has provided valuable information about the relative risk stratification for stage II dMMR CCs. Elevated preoperative CEA and pT4 tumor stage are significantly associated with an increased risk of recurrence. Meanwhile, there is no association between oxaliplatin-based adjuvant chemotherapy and better survival, even in high-risk stage II dMMR CCs. It should be cautioned that the assessment of high-risk factors helps identify patients who are at higher risk of recurrence but does not necessarily mean that they would benefit from oxaliplatin-based adjuvant chemotherapy.

### Supplementary Information

**Additional file 1: Fig S1.** Flow of study.

**Additional file 2: Fig S2.** Assocaition for the clinicopathological risk factior and adjuvant chemotherapy. Blue arrows indicate negative relationships and red arrows indicate positive relationship. Abbreviation: <12 LNs, fewer thsn twelve lymph nodes harvested; LVI, lymphatic vascular invasion; PNI, perineural invasion; Pre-CEA, preoperative carcinoembryonic antigen.

**Additional file 3: Fig S3.** Kaplan-meier plots showing outcome in patients with stage II dMMR colon cancer according to treatment and high-risk factor. (a) Disease-free survival (DFS) in pT4 tumor stage group. (b) DFS in fewer than twelve lymph nodes harvested group. (c) DFS in elevated preoperative CEA group. (d) DFS in elevated postoperative CEA group.

## Data Availability

The datasets used and/or analyzed during the current study available from the corresponding author on reasonable request.

## Abbreviations

CEA: Carcinoembryonic antigen
CCs: Colon cancers
CI: Confidence interval
dMMR: Mismatch repair deficient
DFS: Disease-free survival
FFPE: Formalin-fixed paraffin-embedded
HR: Hazard ritio
IHC: Immunohistochemical
LVI: Lymphatic vascular invasion
OS: Overall survival
PNI: Perineural invasion
