# Supplementary figures and images for: The effects of oxaliplatin-based adjuvant chemotherapy in high-risk stage II colon cancer with mismatch repair-deficient: a retrospective study
Source: BMC Cancer. 2024 Feb 2;24:164. doi: 10.1186/s12885-024-11821-w (PMC10835817; doi:10.1186/s12885-024-11821-w)

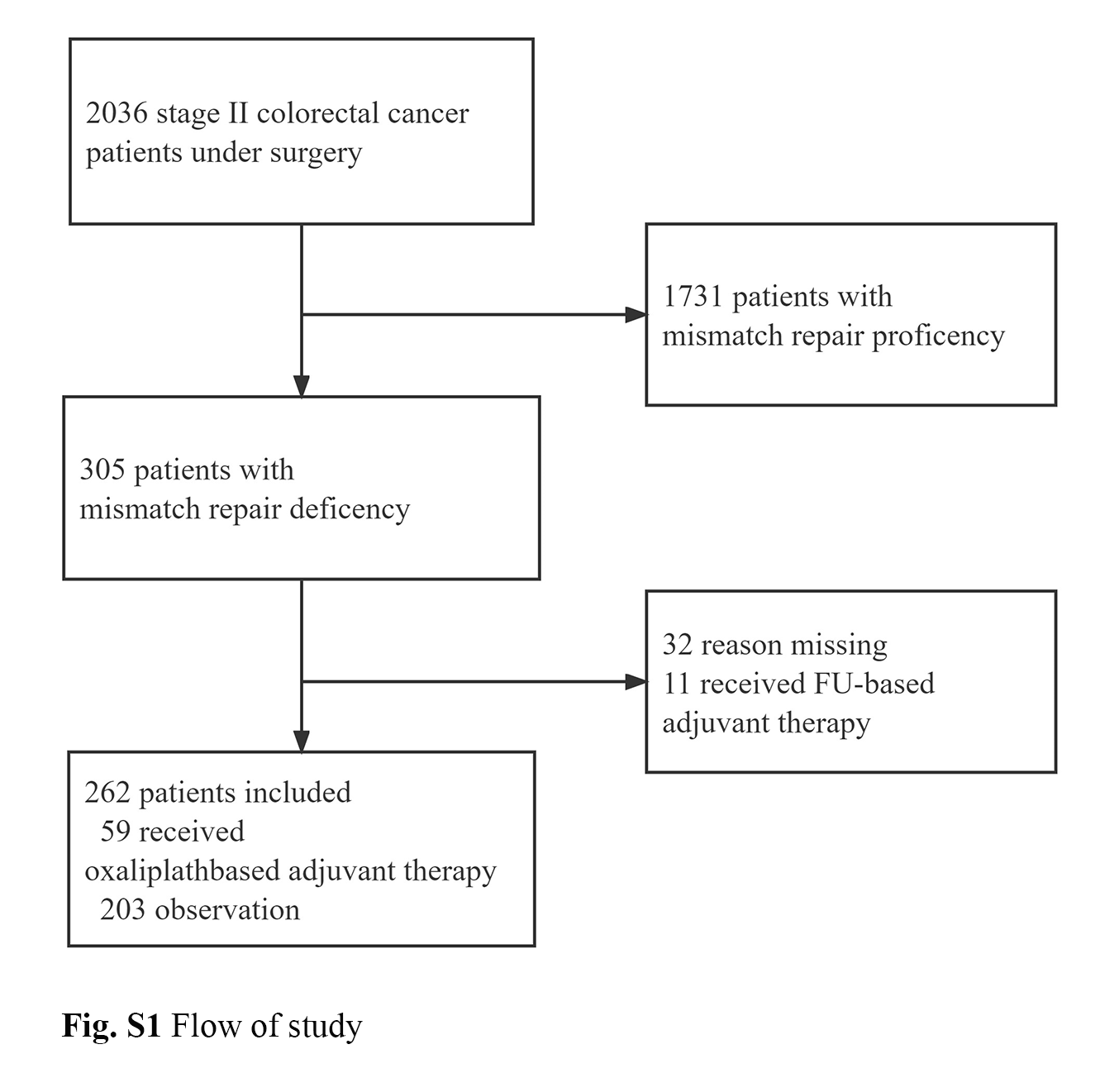

Supplement: Supplementary file 1 — Additional file 1: Fig S1. Flow of study. [file 12885_2024_11821_MOESM1_ESM.tif]

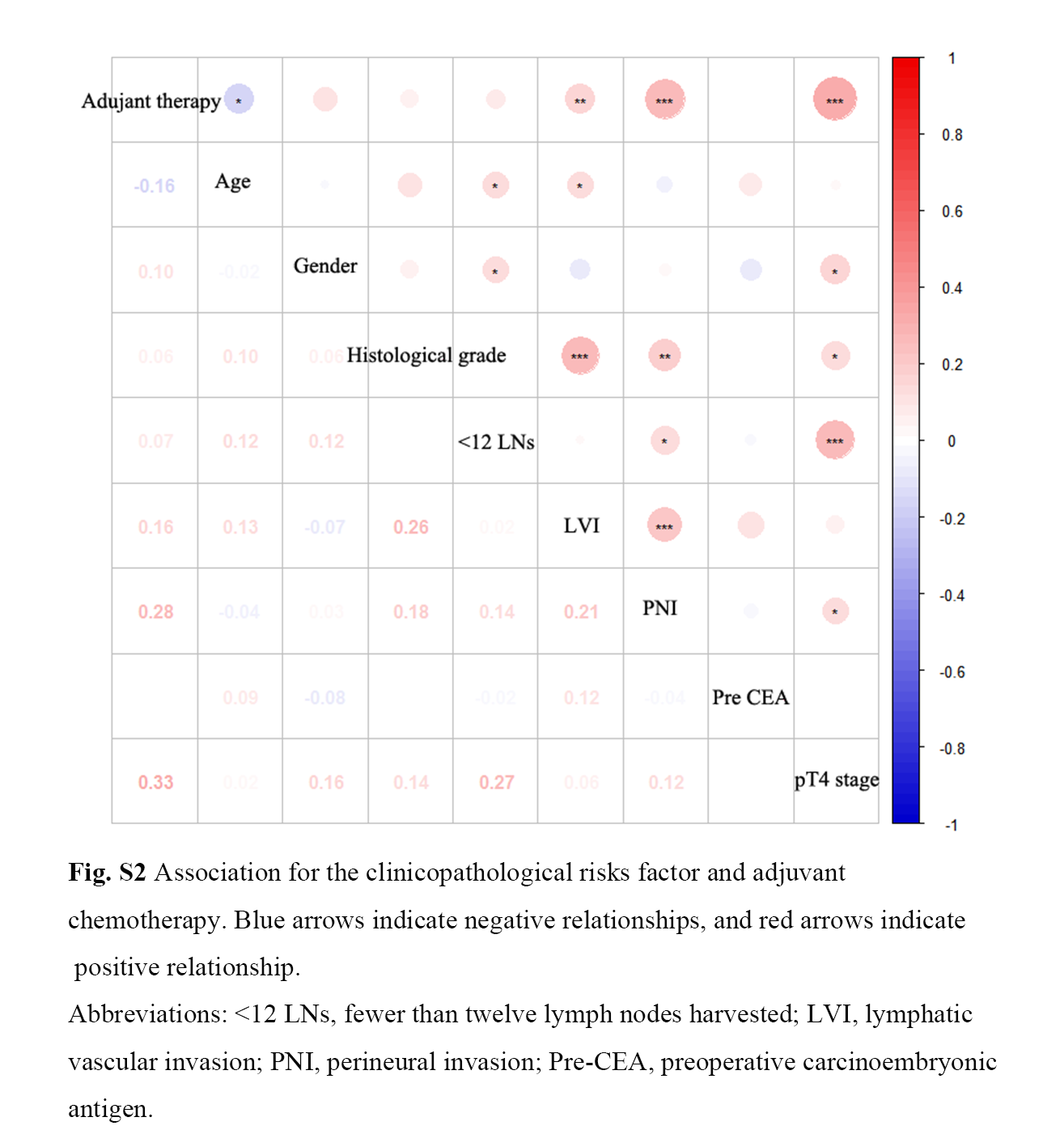

Supplement: Supplementary file 2 — Additional file 2: Fig S2. Assocaition for the clinicopathological risk factior and adjuvant chemotherapy. Blue arrows indicate negative relationships and red arrows indicate positive relationship. Abbreviation: <12 LNs, fewer thsn twelve lymph nodes harvested; LVI, lymphatic vascular invasion; PNI, perineural invasion; Pre-CEA, preoperative carcinoembryonic antigen. [file 12885_2024_11821_MOESM2_ESM.tif]

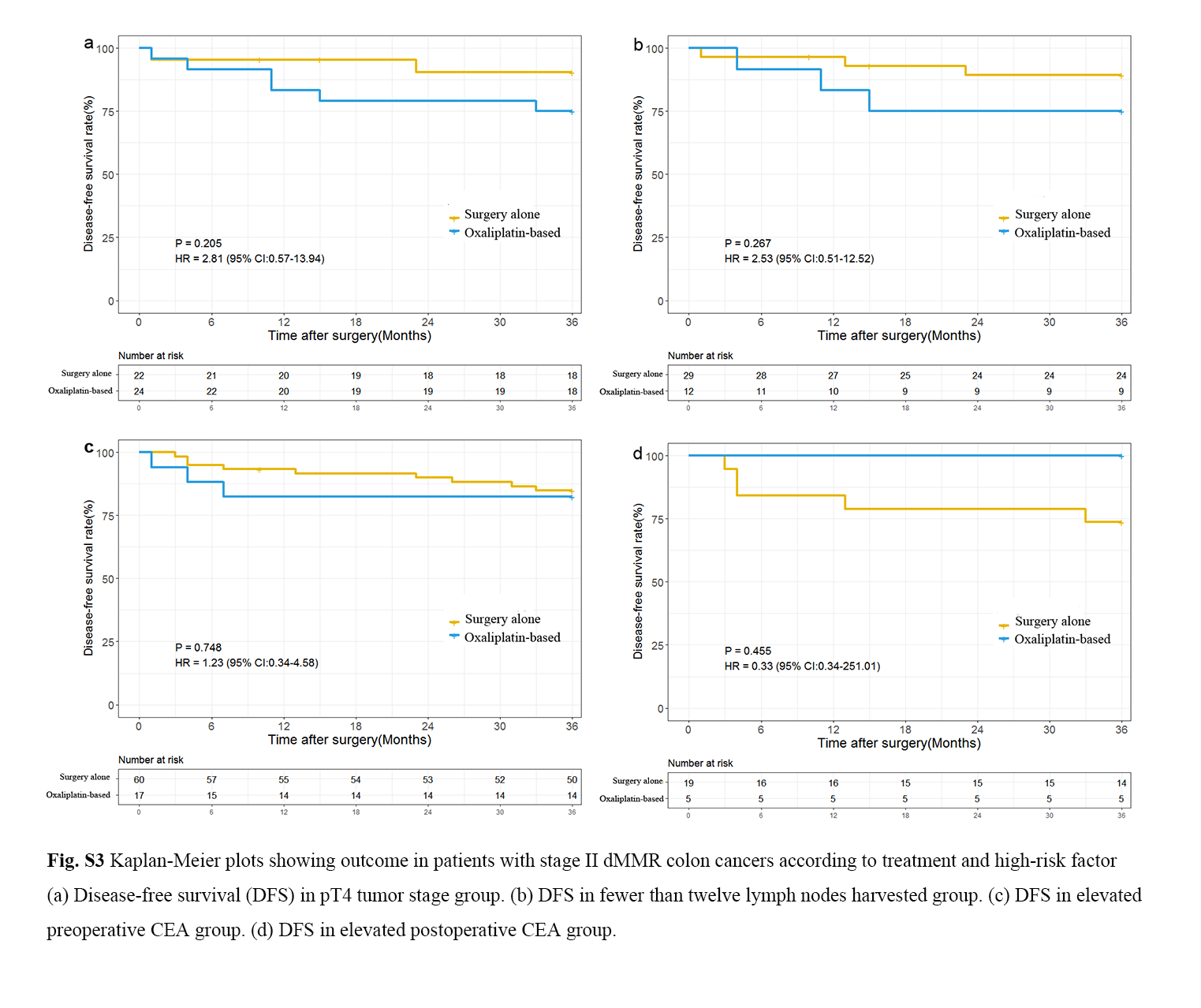

Supplement: Supplementary file 3 — Additional file 3: Fig S3. Kaplan-meier plots showing outcome in patients with stage II dMMR colon cancer according to treatment and high-risk factor. (a) Disease-free survival (DFS) in pT4 tumor stage group. (b) DFS in fewer than twelve lymph nodes harvested group. (c) DFS in elevated preoperative CEA group. (d) DFS in elevated postoperative CEA group. [file 12885_2024_11821_MOESM3_ESM.tif]
